# Frontal cortical control of posterior sensory and association cortices through the claustrum

**DOI:** 10.1007/s00429-018-1661-x

**Published:** 2018-04-06

**Authors:** Michael G. White, Brian N. Mathur

**Affiliations:** Department of Pharmacology, University of Maryland School of Medicine, BRB 4-011, 655 West Baltimore Street, Baltimore, MD 21201 USA

**Keywords:** Anterior cingulate cortex, Visual cortices, Parietal association cortex, Optogenetics, Macrocircuit, Top-down

## Abstract

The claustrum is a telencephalic gray matter nucleus that is richly interconnected with the neocortex. This structure subserves top-down executive functions that require frontal cortical control of posterior cortical regions. However, functional anatomical support for the claustrum allowing for long-range intercortical communication is lacking. To test this, we performed a channelrhodopsin-assisted long-circuit mapping strategy in mouse brain slices. We find that anterior cingulate cortex input to the claustrum is transiently amplified by claustrum neurons that, in turn, project to parietal association cortex or to primary and secondary visual cortices. Additionally, we observe that claustrum drive of cortical neurons in parietal association cortex is layer-specific, eliciting action potential generation briefly in layers II/III, IV, and VI but not V. These data are the first to provide a functional anatomical substrate through claustrum that may underlie top-down functions, such as executive attention or working memory, providing critical insight to this most interconnected and enigmatic nucleus.

## Introduction

Top-down executive processing mediates responses to goal-relevant stimuli that may otherwise lack intrinsic salience. This control engages frontal cortices, the activity of which precedes that of more posterior telencephalic sensory and sensory association cortices (Buschman and Miller 2007; Gregoriou et al. 2009; Miller and Buschman 2013). This phenomenon is causally supported by non-invasive brain activation methods (Ruff et al. 2006; Morishima et al. 2009) and further supported by functional imaging studies that reveal the existence of functionally connected networks involving, for example, frontal and posterior parietal cortical regions during task performance (Corbetta et al. 1993; Kastner and Ungerleider 2000; Corbetta and Shulman 2002). Direct cortico-cortical and cortico-thalamo-cortical circuits are the canonical pathways proposed to underlie the functional linkage of frontal and posterior cortices (Saalmann and Kastner 2011; Miller and Buschman 2013; Buschman and Kastner 2015). Whether other circuits play a role in this function is unknown.

Recently, the claustrum was proposed to interconnect frontal areas with posterior cortices (Mathur 2014). The claustrum is anatomically characterized by widespread connectivity with cortex (Crick and Koch 2005; Mathur 2014). Executive cortices such as the anterior cingulate cortex (ACC) are heavily interconnected with claustrum (Smith and Alloway 2010; White et al. 2017; Wang et al. 2017; Atlan et al. 2017). In addition, activation of afferents from ACC, but not sensory cortices, drives action potential firing of claustrum spiny projection neurons (White et al. 2018). Moreover, ACC input to claustrum is critical for optimal performance on a task that recruits top-down processing (White et al. 2018). These data support a model in which claustrum processes frontal cortical input and may propagate processed signals to posterior sensory and association cortices to mediate top-down executive function (Smith and Alloway 2014). To test if such top-down circuits exist through claustrum, we performed functional mapping of long-range ACC circuits through the claustrum to posterior cortices, specifically ACC → claustrum → visual cortices (V1/V2) and ACC → claustrum → parietal association cortex (PtA) in mouse.

## Materials and methods

### Animals

23 C57BL/6J wildtype mice of both sexes were used. Mice were 10–30 weeks of age at the time of experiments and group-housed with food and water available *ad libitum*. Mice were on a 12-h light–dark cycle beginning at 0700. This study was performed in accordance with the National Institutes of Health Guide for Care and Use of Laboratory Animals and the University of Maryland, School of Medicine, Animal Care and Use Committee.

### Neuronal tract tracing, viral vectors and stereotaxic procedures

For neuronal tract tracer and viral injections, mice were anesthetized via inhalation of 3% isoflurane before being placed in a stereotaxic frame and anesthesia was maintained with inhalation of 1% isoflurane. A small craniotomy was performed over the brain area of interest. For anterograde injections, 100 nL of a 15% solution of 10,000 MW biotinylated dextran amine (BDA; Thermo Fisher Scientific, Waltham, MA) was pressure injected unilaterally into the ACC. For retrograde injections, 100 nL of a 1% solution of cholera toxin B subunit conjugated to AlexaFluor^®^-555 (CTb-555; Thermo Fisher Scientific) was pressured injected unilaterally into the cortical area of interest (V1/V2 or PtA). For macrocircuit mapping of ACC → claustrum → PtA and ACC → claustrum → V1/V2, 150–185 nL of an adeno-associated virus (AAV) vector expressing channelrhodopsin-2 H134R mutation (ChR2) under the *hSyn* promoter with an eYFP fluorescent tag (AAV5-hSyn-ChR2-eYFP; University of Pennsylvania Vector Core) was injected bilaterally at two rostrocaudal levels of the ACC (four total injections). In addition, these mice were also injected bilaterally with 125 nL of the retrogradely transported form of BDA conjugated with Texas Red^®^ (3000 MW; Thermo Fisher Scientific) bilaterally into either V1/V2 or PtA. For functional mapping of claustrum afferents in PtA, 90–120 nL of the AAV-hSyn-ChR2-eYFP construct was injected bilaterally at two rostrocaudal levels of the claustrum (four total injections). For retrograde and anterograde tracer experiments, brains were sectioned 1 week after injection. Viral constructs were allowed to incubate for at least 4 weeks to allow adequate expression of ChR2 before mice were used for slice electrophysiology.

Relative to bregma (dorsal–ventral coordinates were all measured from the brain surface), the coordinates for ACC viral injections were (1) anterior (A)-posterior (P): + 1.34 mm, medial (M)-lateral (L): ± 0.3 mm, dorsal (D)-ventral (V): − 1.25 mm; and (2) A–P: + 0.74 mm, M–L: ± 0.3 mm, D–V: − 1.00 mm. For BDA injections into ACC, a coordinate intermediate to these two described for viral injections was chosen. The coordinates for V1/V2 injections were A–P: − 2.70 mm, M–L: ± 2.05 mm, D–V: − 0.40 mm, and coordinates for PtA injections were A–P: − 1.94 mm, M–L: ± 1.40 mm, D–V: − 0.40 mm. The coordinates for claustrum viral injections were (1) anterior–posterior: + 1.34 mm, medial–lateral ± 2.3 mm, dorsal–ventral (from the brain surface): − 2.35 mm; and (2) anterior–posterior: + 0.86 mm, medial–lateral ± 2.75 mm, dorsal–ventral (from the brain surface): − 2.55 mm.

### Histochemistry and immunohistochemistry

Mice were transcardially perfused with room temperature 0.1 M phosphate-buffered saline (PBS), pH 7.2–7.4, followed by ice-cold 4% (weight/volume) paraformaldehyde in PBS. After the brain was extracted, brains were post-fixed with 4% paraformaldehyde in PBS overnight at 4 °C. Coronal sections were sliced using an Integraslice 7550 MM vibrating microtome (Campden Instruments, Loughborough, England) at a thickness of 50 µm. Sections were either immediately processed or were stored at − 20 °C in a solution of 30% sucrose and 30% ethylene glycol in 0.1M PBS. A conventional protocol was used to visualize anterogradely transported BDA (Mathur and Deutch 2008), which entailed incubation with streptavidin protein conjugated with AlexaFluor^®^-488 (1:1000; Jackson ImmunoResearch, West Grove, PA). Sections from brains injected with CTb were imaged immediately after extraction and sectioning. For post hoc immunohistochemistry of acute brain slices used in whole-cell electrophysiology, Brain BLAQ, a specialized protocol to minimize lipid and aldehyde auto-fluorescence, was used (Kupferschmidt et al. 2015). A chicken anti-GFP (1:2000; Abcam, Cambridge, UK) antibody was used for ChR2-eYFP immunohistochemistry. Secondary anti-chicken antibody conjugated to or AlexaFluor^®^-488 or -555 were used at 1:500 dilutions (Jackson ImmunoResearch). For immunohistochemistry of PtA sections, streptavidin protein conjugated with AlexaFluor^®^-488 (1:1000; Jackson ImmunoResearch) was used to identify PtA neurons that were recorded. The nuclear stain 4′,6-diamidiono-2-phenylindole (1 µg/ml; Sigma-Aldrich, St. Louis, MO) was used to delineate cortical layers.

### Ex vivo brain slice preparation for electrophysiology

Brain slices were prepared according to previously established methods (Mathur et al. 2013). Following deep anesthetization, mice were decapitated and 250 µm thick coronal sections were made using a vibrating microtome in an ice-cold, carbogen (95% O_2,_ 5% CO_2_)-bubbled, high-sucrose artificial cerebrospinal fluid (aCSF). This solution consisted of 194 mM sucrose, 30 mM NaCl, 4.5 mM KCl, 1 mM MgCl_2_, 26 mM NaHCO_3_, 1.2 mM NaH_2_PO_4_, and 10 mM d-glucose. Subsequently, sections were incubated for 30 min at 33 °C in carbogen-bubbled aCSF (315–320 mOsm) which contained 124 mM NaCl, 4.5 mM KCl, 2 mM CaCl_2_, 1 mM MgCl_2_, 26 mM NaHCO_3_, 1.2 mM NaH_2_PO_4_, and 10 mM d-glucose. Sections were incubated at room temperature until use for whole-cell patch-clamp recordings, which were performed in the same aCSF formulation used for incubation.

### Whole-cell current and voltage-clamp recordings

Whole-cell recordings were performed at 29–31 °C using borosilicate glass recording pipettes of 3–7 MΩ resistance. Recording pipettes were filled with a potassium-based solution (290–295 mOsm; pH 7.3) composed of 126 mM potassium gluconate, 4 mM KCl, 10 mM HEPES, 4 mM ATP-Mg, 0.3 mM GTP-Na and 10 mM phosphocreatine. Claustrum neurons projecting to V1/V2 or PtA were targeted for recordings using epifluorescence. For recordings of PtA cortical neurons, pipettes were filled with 3–5% neurobiotin to allow for post hoc identification of neuronal sub-type and the layer from which the recording was performed. Clampex software (version 10.4; Molecular Devices; Sunnyvale, CA) was used for electrophysiological recordings, which were filtered at 2 kHz and digitized at 10 kHz. Optogenetic stimulation was delivered with a 470 nm LED to field illuminate the slice. Individual light pulses in the 20 Hz light trains lasted 3–5 ms, and light trains lasted up to 5 s. Light trains were repeated for 5–10 cycles every 20 s.

### Statistical analyses

To assess the transformation of ACC input that V1/V2- and PtA-projecting claustrum neurons perform, the instantaneous frequency of any action potential bursts was averaged with the inter-pulse frequency (e.g., 20 Hz) at every light pulse. This average frequency was averaged at each pulse across all neurons that fired any action potential in response to ACC stimulation. An exponential function was fit to the average frequency over time using GraphPad Prism (version 6.0.1; GraphPad Software; La Jolla, CA). For responsivity analyses of the various circuits tested, the percentage of neurons that did not exhibit excitatory post-synaptic potentials or action potential generation, the percentage of neurons that exhibited excitatory post-synaptic potentials without action potential generation, and the percentage of neurons that fired action potentials were determined. In addition, the percentage of pulses that elicited an action potential was calculated over the first 1 or 2 s of the 20 Hz light train for neurons that fired action potentials. Each action potential in a burst was counted toward this metric; as such, percentages over 100% were possible for this metric.

## Results

### Claustrum anatomy supports long-range connectivity from ACC to posterior cortices

**Fig. 1:**
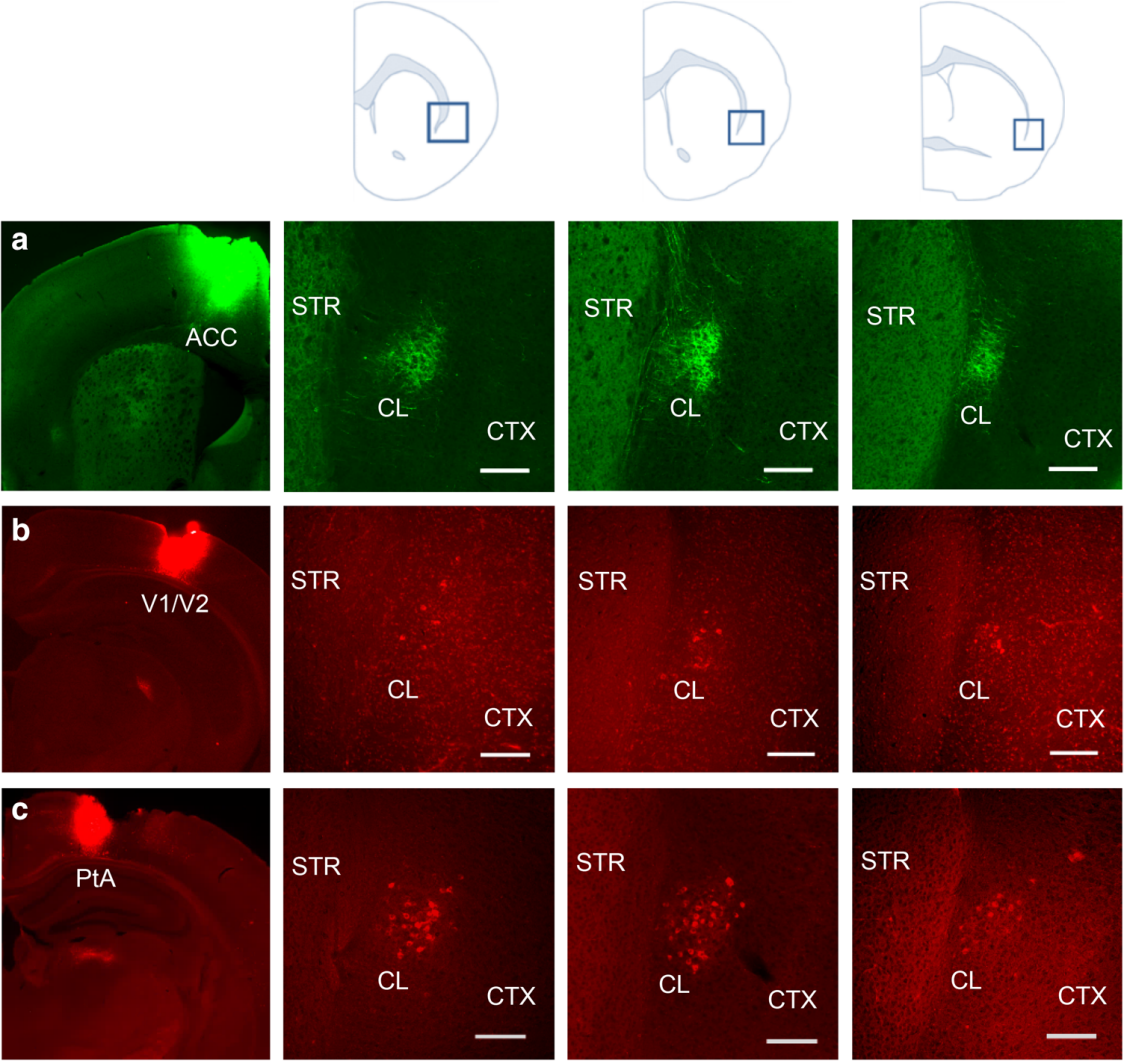
Claustrum receives dense innervation from anterior cingulate cortex (ACC) and projects to visual cortices (V1/V2) and parietal association cortex (PtA). **a** Left: photomicrograph showing anterograde neuronal tract tracer biotinylated dextran amine (BDA, 10,000 MW; green) injected into ACC. Right: BDA-labeled terminals (green) from ACC densely innervated contralateral claustrum at all three rostrocaudal levels of claustrum as depicted by the blue boxes in the anatomical cartoons above. **b** Left: photomicrograph showing retrograde tract tracer cholera toxin B subunit (CTb; red) injected into V1/V2. Right: CTb-labeled cells projecting to V1/V2 were found at all three rostrocaudal levels of ipsilateral claustrum. **c** Left: photomicrograph showing CTb (red) injected into PtA. Right: a dense population of CTb-labeled cells projecting to PtA were found at all three rostrocaudal levels of ipsilateral claustrum. *STR* striatum, *CL* claustrum, *CTX* cortex. Scale bars—200 µm

We first examined whether claustrum contains the necessary circuitry for relaying ACC input to posterior cortices responsible for visuospatial processing, including primary and secondary visual (V1/V2) and parietal association cortex (PtA). This portion of parietal cortex, analogous to posterior parietal cortex in the primate, encodes higher order functions, such as decision-making and navigation (Kolb and Walkey 1987; Torrealba and Valdés 2008; Harvey et al. 2012). It is anatomically distinct from neighboring somatosensory cortex (Reep et al. 1994). ACC inputs to the claustrum were identified using an injection of an anterograde tracer injection into ACC (*n* = 3), which revealed dense terminal labeling in the contralateral claustrum across its rostral-caudal extent (Fig. 1a). In separate mice, we injected a retrograde tract tracer into two areas along the visual processing hierarchy: V1/V2 or PtA. Retrograde tracer injection into V1/V2 (*n* = 3) labeled a population of claustrum neurons at each level of ipsilateral claustrum across its rostral-caudal axis (Fig. 1b). Retrograde tracer injection into PtA (*n* = 3) also revealed tracer-filled claustrum projection neurons at all levels of ipsilateral claustrum (Fig. 1c), which were more numerous than projections to V1/V2 (Fig. 1b). Dense contralateral inputs to and primarily ipsilateral outputs from claustrum is a consistent connectivity pattern in rodent (Smith and Alloway 2010; Mathur 2014).

### Claustrum propagates ACC input to PtA and V1/V2

**Fig. 2:**
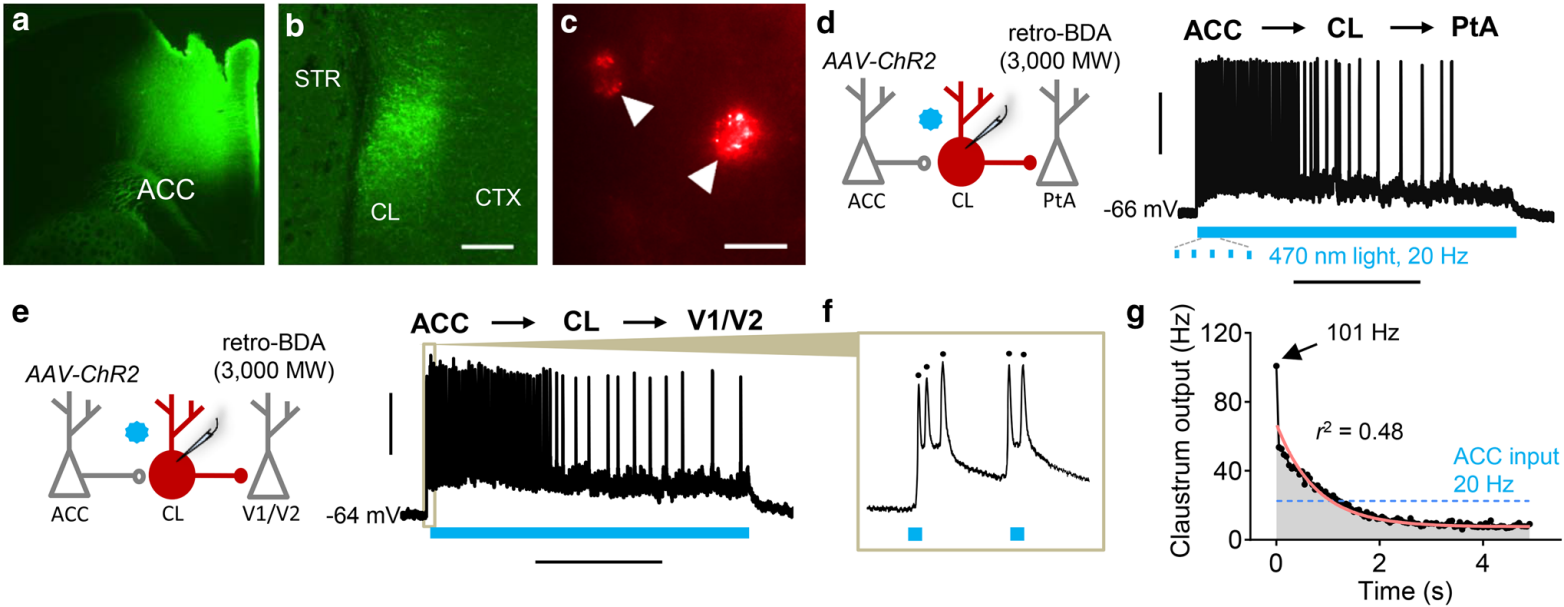
Claustrum neurons projecting to PtA and V1/V2 receive and transiently amplify ACC input. **a** Photomicrograph showing adeno-associated virus expressing channelrhodopsin-2 (AAV-ChR2) with eYFP tag injected into ACC. **b** ACC afferents expressing ChR2 (green) densely labeled the claustrum. **c** PtA projection neurons in claustrum (arrows) were labeled with retrogradely trafficked BDA (retro-BDA; 3000 MW) that was injected into PtA. **d** Left: ChR2-assisted macrocircuit mapping strategy entailed optogenetic stimulation of ACC afferents (AAV-ChR2) with 470 nm light (blue star) while performing whole-cell recordings from claustrum (CL) projection neurons to PtA. Right: representative trace showing a claustrum neuron projecting to PtA that initially burst-fired in response to a 5 s train of 20 Hz optogenetic ACC stimulation. Firing diminished as the stimulation train progressed. **e** The same macrocircuit mapping strategy as in **d** was employed to target claustrum neurons projecting to V1/V2 while recording responses to ACC afferent stimulation. Right: representative trace showing a claustrum neuron projecting to V1/V2 that initially burst-fired in response to a 5 s train of 20 Hz ACC stimulation. Firing diminished over time. **f** Representative trace showing a V1/V2-projecting neuron firing multiple action potentials in response to single pulses of ACC afferent stimulation. **g** Graph showing average output of V1/V2- and PtA-projecting claustrum neurons over time in response to a constant ACC input (20 Hz for 5 s). Peak average output was at the first light pulse (101 Hz) and exponentially decayed over the light train (*n* = 15; *r*^2^ = 0.48, *p* < 0.005). Horizontal scale bars—200 µm **b**; 25 µm **c**; 2 s **d, e**. Vertical scale bars—30 mV

Our tracing experiments show that the mouse claustrum receives a dense ACC input and that the claustrum projects to V1 and PtA. However, it is unclear if the claustrum is functionally capable of propagating information arising from the ACC to visual cortices. To test this, we employed a channelrhodopsin-2 (ChR2)-assisted macrocircuit mapping strategy where we injected an adeno-associated virus expressing ChR2 (AAV-ChR2) into the ACC. This injection resulted in dense labeling of ACC afferents in the claustrum expressing fluorescently labeled ChR2 (Fig. 2a, b). In the same animals injected with AAV-ChR2 in ACC, we injected a retrograde tract tracer (retro-BDA, 3000 MW) into either V1/V2 or PtA. The retrograde tracer injections allowed us to visually identify claustrum neurons projecting to either V1/V2 or PtA (Fig. 2c) and target them for whole-cell patch-clamp recordings in acute mouse brain slices. To test if these different projection neurons propagated ACC input, we delivered 470 nm light for 5 s at 20 Hz to activate ACC terminals while recording the retro-BDA-labeled claustrum neurons in a current clamp configuration. 20 Hz optogenetic activation of ACC afferents in the claustrum drove claustrum neurons projecting to V1/V2 and PtA to fire action potentials (Fig. 2d, e). These neurons also burst-fired in response to single 470 nm light pulses early in the 20 Hz train (Fig. 2f) and exhibited habituation as the train progressed (Fig. 2d, e). We plotted the instantaneous change in neuronal output for each light pulse (Fig. 2g) and observed that the effective claustrum output peaked on the first pulse at 101 Hz and exponentially declined during the stimulation. A summary of the responsivity to ACC afferent stimulation of claustrum neurons projecting to either V1/V2 or PtA is shown in the first two rows of Table 1.

**Table 1:** Summary of long-range macrocircuit and CL → PtA circuit mapping

Circuit	% unresponsive	% subthreshold	% firing	% AP/light pulses	*n*
ACC → CL → V1/V2	0	0	100	88.6^a^	9
ACC → CL → PtA	11.1	22.2	66.7	53.3^a^	9
CL → PtA (II/III)	54.5	0	45.5	17.2^b^	11
CL → PtA (IV)	50	0	50	6.2^b^	10
CL → PtA (V)	50	50	0	N/A	10
CL → PtA (VI)	50	0	50	13.9^b^	14

For each circuit investigated the percentage of unresponsive neurons (% unresponsive), percentage of neurons exhibiting subthreshold responses (% subthreshold), percentage of neurons that fired an action potential (AP; % firing), the percentage of light pulses that elicited an AP for firing neurons (% AP/light pulses), and the number of cells recorded (*n*) is given. For CL → PtA circuits, the layer of PtA is noted in parentheses

^a^20 Hz for 2 s

^b^20 Hz for 1 s

### Claustrum provides brief excitatory drive layer-specifically across PtA

**Fig. 3:**
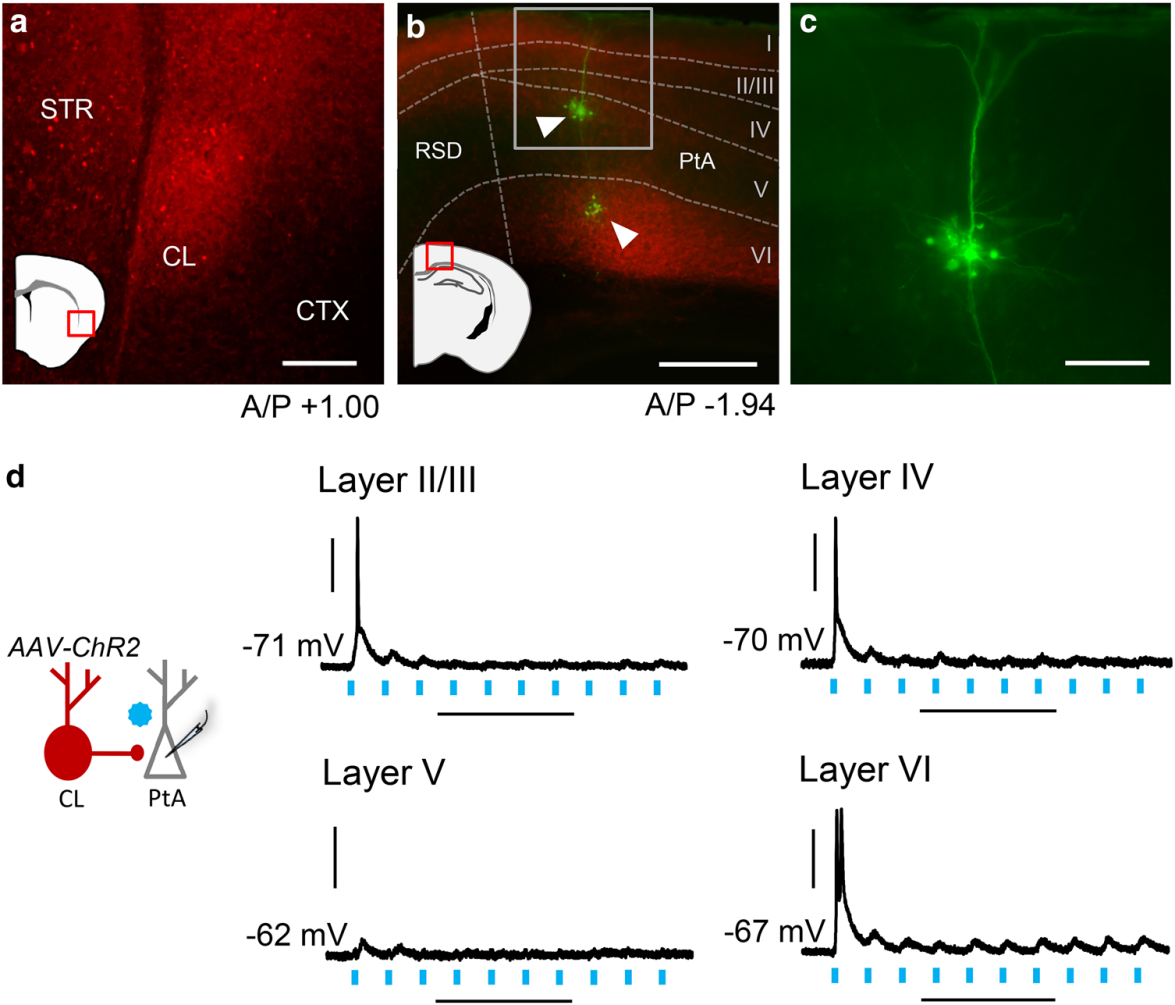
Claustrum afferent stimulation drives PtA neuron firing in layers II/III, IV, and VI but not V. **a** Photomicrograph showing AAV-ChR2 (red) expression after injection into the claustrum. The photomicrograph location is indicated by the red box on the cartoon. **b** Photomicrograph showing PtA layer-specific innervation of claustrum afferents expressing ChR2 (red box shows PtA inset region). Neurons across PtA layers were targeted for whole-cell recordings and filled with neurobiotin (green, arrowheads). Cortical layers were delineated by staining with 4′,6-diamidine-2′-phenylindole (DAPI, not shown). Innervation of layer V was relatively sparse compared to other cortical layers. **c** Inset from **b** showing PtA layer V pyramidal neuron at high magnification. **d** Left: schematic showing claustrum innervation of PtA layers. Right: representative traces showing responses to optogenetic stimulation of claustrum afferents (20 Hz, 470 nm light). Action potential firing was noted at initial light pulses across PtA layers except for layer V. Horizontal scale bars—200 µm **a**; 400 µm **b**; 100 µm **c**; 200 ms **d**. Vertical scale bars—30 mV. *A/P* anterior/posterior, *RSD* retrosplenial dysgranular cortex

Our functional macrocircuit mapping demonstrates that a circuit exists for communication from frontal cortices to V1/V2 and PtA through claustrum. Although claustrum projection neurons are glutamatergic (Brand 1981; Braak and Braak 1982; Hur and Zaborszky 2005; Watakabe et al. 2014; Kim et al. 2016), the downstream impact of claustrum activity on cortical layers in rodents is unclear. As such, we injected AAV-ChR2 into claustrum (Fig. 3a) and performed whole-cell recordings across the layers of PtA while stimulating claustrum afferents. Our recordings were restricted to PtA because of the denser number of claustrum cells projecting to PtA relative to V1/V2 (see Fig. 1). Neurons were filled with neurobiotin to allow for post hoc discrimination of pyramidal neurons from interneurons, and slices were stained post hoc for ChR2 (Fig. 3b, c). To identify the cortical layers from which neurons were recorded, we used a 4′,6-Diamidino-2-phenylindole (DAPI) stain. Representative responses from each cortical layer are shown in Fig. 3d and summary data are given in the last four rows of Table 1. Notably, we detected AP generation in approximately half of all neurons recorded, excluding those in layer V, which we identified to be pyramidal in all cases (*n* = 10 of 10). All of the neurons recorded from layers II/III were also all pyramidal (*n* = 11 of 11). Neuron sub-types recorded from layers IV and VI were heterogeneous: pyramidal neurons (layer IV: *n* = 1 of 10; layer VI: *n* = 1 of 14), spiny neurons without any detectable apical dendrite (layer IV: *n* = 5 of 10; layer VI: *n* = 4 of 14), and aspiny neurons (layer IV: *n* = 4 of 10; layer VI: *n* = 6 of 14). In layer VI, 3 of 14 neurons recorded were unable to be classified.

## Discussion

In this study, we show that the claustrum provides a functional anatomical link between frontal cortices, specifically ACC, and posterior sensory/association cortices (i.e., V1/V2 and PtA). The intercortical circuit configuration described herein supports a model wherein the claustrum processes and propagates top-down signals arising from frontal cortices to coordinate cortical activity of hierarchically lower cortices (Mathur 2014). In addition, we show that claustrum neurons projecting to V1/V2 and PtA transiently amplify a fixed 20 Hz ACC input and that claustrum briefly drives action potential generation in neurons residing in layers II/III, IV, and VI of PtA. Further work is necessary to determine how both circuit configurations might act in concert to subserve cognition.

A fundamental question regarding claustrum function is how it may be unique from that of associative thalamic nuclei or direct cortico-cortical connections. Thalamic nuclei allow for communication between cortices (Pinault 2004; Saalmann and Kastner 2011; Sherman and Guillery 2011), and areas of pulvinar in particular exhibit connectivity with both parietal and frontal cortices (Gutierrez et al. 2000; Arcaro et al. 2015). Additionally, long-range, direct frontal inputs enhance firing of visual cortical neurons to their preferred orientation (Zhang et al. 2014). This begs the question of why claustra would also exist to link frontal and posterior cortical centers. Direct cortico-cortical inputs arising from ACC predominantly innervate cortical layers II and III of granular cortices (Barbas and Rempel-Clower 1997) and thalamic nuclei, such as pulvinar, primarily innervate layer IV and more superficial layers (Benevento and Rezak 1976; Shipp 2003). In contrast, our findings indicate that claustrum has quite widespread innervation across layers and specifically drives firing of neurons in layers II/III, IV and VI of PtA. While both claustrum and associative thalamic structures, such as the pulvinar nucleus, innervate the cortex and exhibit transient responses to visual stimuli in monkeys (Remedios et al. 2010; Petersen et al. 1985), how these structures innervate and influence cortical activity may differ. Our results demonstrate that claustrum transiently drives PtA neuron firing, whereas associative thalamic synapses onto cortical neurons provides a modulatory input in rodents (Cruikshank et al. 2012; Mease et al. 2016). Notably, however, studies examining thalamic afferent drive of specifically parietal cortex neurons are lacking. Thus, the claustrum may provide unique processing and layer-specific innervation compared to the thalamocortical and cortico-cortical connections.

Taken together, the cortico-claustro-cortical circuitry described herein represents a previously unrecognized system in brain positioned to enable spatiotemporal control of distributed cortical sites. While further work is needed to fully understand how claustrum neurons that are driven by the ACC are, in turn, controlling cortical areas such as PtA and V1/V2, the present data provide a novel top-down system that may undergo perturbation in neuropsychiatric illness (Morys et al. 1996; Cascella et al. 2011).
